# Supplementary material for: State-of-the-Art of Profiling Immune Contexture in the Era of Multiplexed Staining and Digital Analysis to Study Paraffin Tumor Tissues
Source: Cancers (Basel). 2019 Feb 20;11(2):247. doi: 10.3390/cancers11020247 (PMC6406364; doi:10.3390/cancers11020247)
Supplement: Supplementary file 1 [file cancers-11-00247-s001.pdf]

Supplementary Materials: State-of-the-Art of Profiling Immune Contexture in the Era of Multiplexed Staining and Digital Analysis to Study Paraffin Tumor Tissues

Edwin Roger Parra, Alejandro Francisco-Cruz, Ignacio Ivan Wistuba

Table S1. Multiplex image acquisition products.

Company	Imaging Modality	Image Acquisition Instrument	Image Extraction	Image Resolution
Olympus	BF	Olympus VS120-L-110W	WS	20× (0.33 μm/pixel) and 40× (0.17 μm/pixel)
Nikon	BF	Eclipse Ci-E	WS	20× (0.32 μm/pixel) and 40× (0.16 μm/pixel)
Keyence	FL	BZ-X710	ROI	40× oil immersion (0.188 μm/pixel)
Hamamatsu	BF, FL	NanoZoomer S60	WS	20× (0.46 μm/pixel), and 40× (0.23 μm/pixel)
Zeiss	BF, FL	AxioVision MosaiX	WS	5× (2.11 μm/pixel), 10× (1.05 μm/pixel), and 20× (0.53 μm/pixel)
Olympus America	BF, FL	VS110	WS	20× (0.46 μm/pixel), and 40× (0.23 μm/pixel)
Akoya /PerkinElmer	BF, FL	Vectra/Polaris	ROI	10× (1.0 μm/pixel), 20× (0.5 μm/pixel), and 40× (0.25 μm/pixel)
Leica Biosystems	FL	Aperio IF	WS	20× (0.25 μm/pixel) and 40× (0.50 μm/pixel)
Ventana/Roche	BF, FL	iScan	WS	4× (2.27 μm/pixel), 20× (0.908 μm/pixel) ,20× (0.454 μm/pixel) and 40x (0.2227 μm/pixel)
3DHistech	BF, FL	Pannoramic/250 FLASH III	WS	30× (0.32 μm/pixel) and 60× (0.16 μm/pixel)
Huron Technologies	BF, FL	TISSUEscope 4000	WS	20× (0.4 μm/pixel) and 40× (0.2 μm/pixel)
MetaSystems	BF, FL	Metafer	WS	20× (0.46 μm/pixel), and 40× (0.23 μm/pixel)
MikroScan Technologies	BF, FL	MikroScan	WS	2× (4.54 μm/pixel), 4× (2.27 μm/pixel), 10× (0.9087 μm/pixel), 20× (0.454 μm/pixel), and 40× (0.227 μm/pixel)
TissueGnostics	BF, FL	TissueFAXS	WS	10× to 100× (4.25μm μm/pixel)
Bruker	Metal tag spectra captured based on mass	MALDI-TOF	ROI	~200 nm
IONPATH	Metal tag spectra captured based on mass	MIBIScope-I	ROI	FOV 1 mm2, ≥1 mm2/2 hr, 250 nm
FLUIDIGM	Metal tag spectra captured based on mass	Helios/CyTOF	ROI	FOV > ≥ 250 μm × 250 μm, ≥1 mm2/2 hr, 200 pixels/sec, 1000 nm

Note: BF = Bright Field, FL = Fluorescence, FOV = Field of view, WS = Whole Section, ROI = Region of Interest.
